# Neuroticism Associated with Cocaine-Induced Psychosis in Cocaine-Dependent Patients: A Cross-Sectional Observational Study

**DOI:** 10.1371/journal.pone.0106111

**Published:** 2014-09-25

**Authors:** Carlos Roncero, Constanza Daigre, Carmen Barral, Elena Ros-Cucurull, Lara Grau-López, Laia Rodríguez-Cintas, Nuria Tarifa, Miguel Casas, Sergi Valero

**Affiliations:** 1 Outpatient Drug Clinic, Department of Psychiatry, Vall d’Hebron University Hospital - Public Health Agency, Barcelona (ASPB), CIBERSAM, Barcelona, Spain; 2 Department of Psychiatry, Vall d’Hebron University Hospital, CIBERSAM, Barcelona, Spain; 3 Department of Psychiatry and Legal Medicine, Universitat Autònoma de Barcelona, Barcelona, Spain; National Cheng Kung University, Taiwan

## Abstract

**Background:**

Cocaine consumption can induce transient psychotic symptoms, which has been correlated with more severe addiction and aggressive behavior. However, little is known about the nature of the relationship between personality traits and psychotic symptoms in cocaine-dependent patients. This study examined the relationship between neuroticism and cocaine-induced psychosis.

**Methods:**

A total of 231 cocaine-dependent patients seeking treatment were recruited to the study. Personality was evaluated by the Zuckerman-Kuhlman Personality Questionnaire. Cocaine-induced psychosis questionnaire, SCID-I, and SCID-II were used to evaluate comorbidity and clinical characteristics. Data analysis was performed in three steps: descriptive, bivariate, and multivariate analyses.

**Results:**

Cocaine-induced psychosis was reported in 65.4% of the patients and some personality disorder in 46.8%. Two personality dimensions (Neuroticism-Anxiety and Aggression-Hostility) presented a significant effect on the risk of experiencing psychotic symptoms (*t*(229) = 2.69, *p* = 0.008; *t*(229) = 2.06, *p* = 0.004), and patients with psychotic symptoms showed higher scores in both variables. On the multivariate analysis, only Neuroticism remained as a significant personality factor independently associated with psychotic symptoms (Wald = 7.44, *p*<0.05, OR = 1.08, CI 95% 1.02–1.16) after controlling for age, gender and number of consumption substances.

**Conclusions:**

An association between high neuroticism scores and presence of psychotic symptoms induced by cocaine has been found, independently of other consumption variables. Personality dimensions should be evaluated in cocaine-dependent patients in order to detect high scores of neuroticism and warn patients about the risk of developing cocaine-induced psychotic symptoms.

## Introduction

Cocaine consumption and demands for treatment have increased in Europe and USA [1]. The US National Comorbidity Survey found that 3.8% of the general population has a substance use disorder [2], [3]. The number of cocaine users entering treatment for the first time increased from 35,000 patients in 2006 to 37,000 in 2009 and then declined to 31,000 in 2011. In Europe, it is estimated that about 1.9% of young adults used cocaine in the past year, and this rate is even higher in Spain [4]. The rates of consumption in European general population vary between 0.2% and 0.5%. It is used predominantly in Spain, UK, Germany, Italy and the Netherlands [5].

Cocaine consumption can induce transient psychotic symptoms, expressed as paranoia or hallucinations [6]. The term cocaine-induced psychosis (CIP) has been used to describe this syndrome [7]–[10], which tends to appear after cocaine use [11], [12], with transitory paranoia being the most common symptom [6], [13]–[15]. This phenomenon has been described in different studies in the U.S. [7]–[9] and more recently, in Europe [6], [13], [15]–[17]. In cocaine dependent patients, CIP is highly prevalent across the lifespan (among 60.0%–86.5%) [6], [13], [15], [18] but the presence of psychotic symptoms is not a universal feature. Although many risk factors of CIP in cocaine-dependent patients have been described, they have yet to be conclusively elaborated [18]. CIP has been associated with greater addiction severity [19], [20] agitated behavior, and aggression [12], [15]. Although assessment of personality during heavy drug use is complex, and there is some debate about its stability [16], [21], [22] antisocial personality disorder has been proposed as a risk factor for CIP [13]. However, not all studies have found this association [9], [23]. As this relationship is controversial, the personality dimension approach could be proposed in order to clarify the relationship between personality and CIP.

Differences have been described in the personality traits of individuals associated with and without drug dependence [24], including those related to cocaine dependence [25] Recently, differences have been observed in the personality traits of cocaine-dependent patients with or without CIP [26].

Neuroticism is a fundamental dimension of personality, as established in numerous factor analytic studies involving diverse sources of data [27]–[29]. The correlates of neuroticism have been increasingly investigated, and they suggest broad dysfunction. Neurotic individuals are both self-critical [30] and have hostile thoughts concerning others [31]. They are prone to a wide variety of negative emotional experiences, whether related to purportedly basic emotions [32] or to self-conscious emotions [33]. Neurotic individuals report greater impulsivity and poorer self-control [34]. Neuroticism has been considered a major personality domain of public health interest with important associations with mental disorders [35]. Higher levels of neuroticism predispose individuals to personality and substance use disorders [36]. Neuroticism has been identified as one of the most robust factors characterizing the drug-dependent population [37], [38]. This relationship is consistent using the Zuckerman-Kuhlman Personality Questionnaire (ZKPQ) [24], the Eysenk Personality Profile (EPQ) [25], or the NEO-PI [26], [39].

Cocaine-dependent patients score high in neuroticism [40]. Furthermore, anxious-impulsive personality traits may represent endophenotypes associated with the risk of developing cocaine dependence [41]. As well, in cocaine-dependent patients the Neuroticism-Anxiety scale was associated with greater drug abuse and psychiatric severity and worse outcome [42].

The relationship between neuroticism and psychotic symptoms, even the severity of these symptoms, has been studied previously in non-drug using populations [43]. Moreover, non addicted patients in their first psychotic episode, scored higher in neuroticism than healthy participants [44], [45].

Considering the above, it is expected that neuroticism increases the vulnerability of induced psychosis; therefore, the effect of neuroticism on the risk of experiencing induced psychosis should be stronger among cocaine-dependent patients with high neuroticism scores.

To our knowledge, there is very little research on the relationship between CIP and neuroticism in cocaine-dependent patients. Therefore, the aim of this study was to examine the relationship between CIP and personality trails evaluated with the ZKPQ, in an attempt to determine whether personality dimensions are associated with CIP.

## Materials and Methods

### Participants

**Figure 1:**
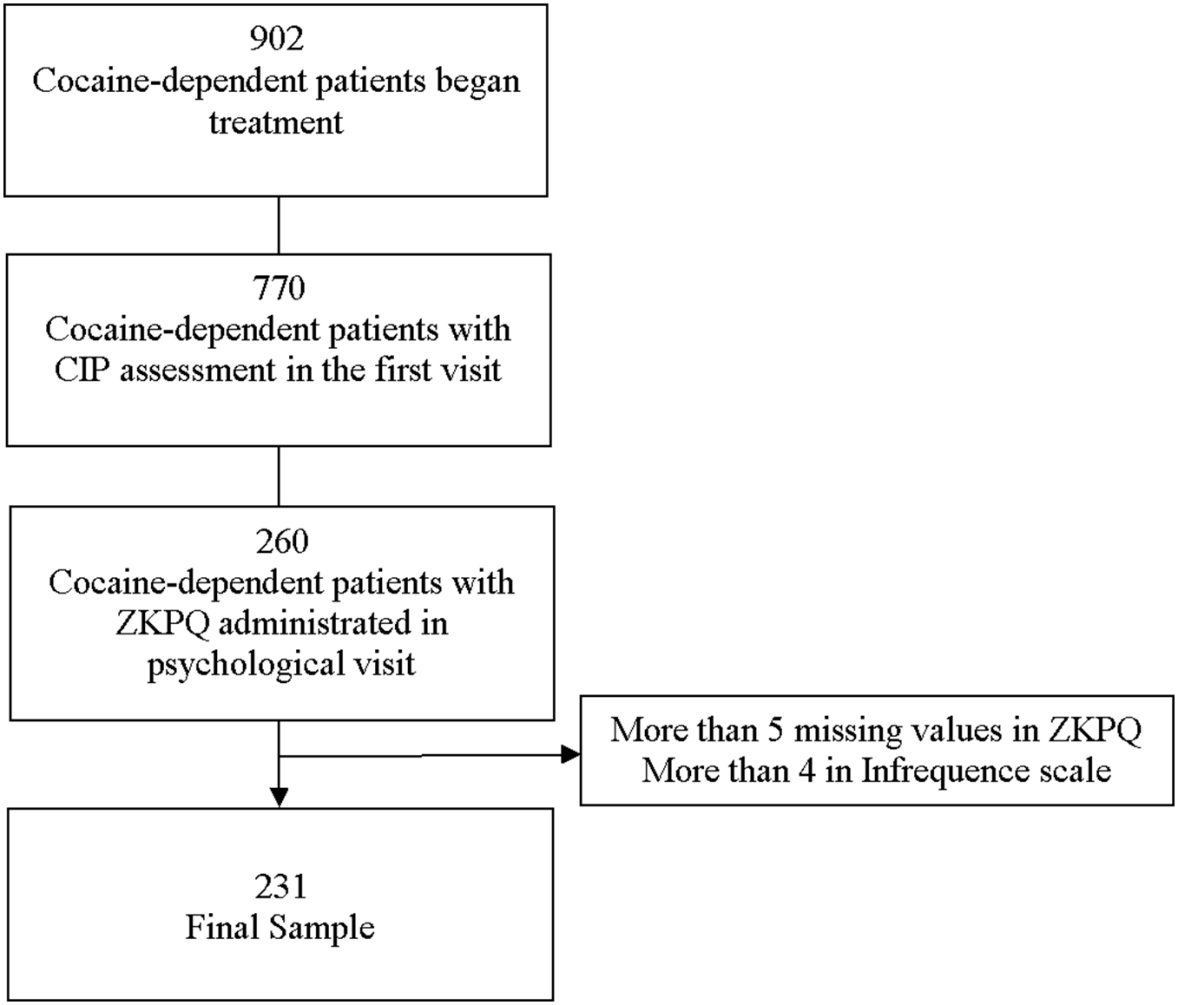
Study Flow Chart.

From a total of 902 patients seeking treatment for cocaine-dependence in our unit, 231 (77.5% males, mean age 36, 33 years old, range: 19–59) took part in the study. The flow chart of the study is presented in Figure 1.

We performed a cross-sectional, observational study. The participants were cocaine-dependent patients, diagnosed according to the *Diagnostic and Statistical Manual of Mental Disorders* (DSM-IV-TR) [46]; seeking treatment in the Drug Unit of the Psychiatric Department of the Vall d’Hebron Hospital (Barcelona, Spain). They began treatment between May 2009 and June 2013. This study is part of a more extensive research on comorbidity in cocaine dependence.

Inclusion criteria: being over 18 years old, cocaine dependence according to DSM-IV criteria, and signing the informed consent prior to participation. Exclusion criteria: psychotic disorder or bipolar Type I disorder, intoxication or psychotic states at the examination, severe somatic disease at baseline examination, and low Spanish language proficiency. Psychotic and bipolar patients were excluded in order to avoid the risk of being unable to distinguish primary and secondary symptomatology. The research was approved by the Ethics Committee of the Vall d’Hebron Hospital. Patients did not receive any financial compensation for taking part in this study.

### Measures

Cocaine Induced Psychosis (CIP) was evaluated by trained psychiatrists who systematically conducted a previously described structured interview [13]. There was a systematic register that recorded the variables related to cocaine consumption, such as the amount of cocaine consumed per week, years of cocaine dependence, and main cocaine route of administration at the beginning of the treatment.

In order to evaluate substance use disorders, the Structured Clinical Interview for DSM IV Axis I disorders (SCID I) was used [47]. This tool is widely used and has shown good psychometric properties [48]. The Structured Clinical Interview for DSM IV Axis II Personality Disorders (SCID II) was used to evaluate the presence personality disorders. This tool has shown adequate reliability and usefulness in providing fine discriminations between the different Axis II disorders [49]. The Spanish translation has shown good psychometric properties [50].

Personality Traits: The Zuckerman-Kuhlman Personality Questionnaire (ZKPQ) [51] was used to assess personality. It consists of five scales: 1. Neuroticism-Anxiety (N-Anx, 19 items) items describe frequent emotional upset, tension, worry, fearfulness, obsessive indecision, lack of self-confidence, and sensitivity to criticism. 2. Activity (Act, 17 items) items describe the need for general activity, an inability to relax and do nothing when the opportunity arises, a preference for hard and challenging work, an active busy life, and high energy level. Two facet scores can be obtained from this scale: Need for General Activity, impatience and restlessness (GenAct, 9 items) and need for Work Activity (WorkAct, 8 items). 3. Sociability (Sy, 17 items) items describe the number of friends one has and the amount of time spent with them, outgoingness at parties, and a preference for being with others as opposed to being alone and engaging in solitary activities. Two facet scores can also be obtained: Parties and friends (Parties, 9 items) and Isolation Intolerance (Isol, 8 items). 4. Impulsive Sensation-Seeking (ImpSS, 19 items) items involve a lack of planning and the tendency to act without thinking, and seeking excitement novel experiences, and a willingness to take risks for these types of experiences. The ImpSS items are general in content and do not describe specific activities such as risky sports, drinking, having sex or drug consumption. Two facet scores can be obtained from this scale: Impulsivity (Imp, 8 items) and Sensation Seeking (SS, 11 items). 5. Aggression-Hostility (Agg-Host, 17 items) items describe a readiness to express verbal aggression; rude, thoughtless or antisocial behavior; vengefulness and spitefulness; having a quick temper and impatience with others. Participants of this study completed the Spanish version of the ZKPQ [52]. The goodness of its psychometric properties have been tested not only in drug-dependence [24] but also in other clinical samples [53]–[55]. In this sample, Cronbach’s alpha of ZKPQ scales were: ImpSSs: .82, N_Anx: .86, Agg-Host: .70, Act: .70 and Sy: .76.

### Procedure

The evaluation process consisted of three interview sessions conducted by trained psychiatrists and psychologists. During the first medical visit, the psychiatrists performed the evaluation of substance use disorders, CIP, and variables related to cocaine consumption. Psychologists measured the severity of substance use disorder and personality traits in the second and third interviews.

### Data Analysis

Data analysis was performed in three steps: descriptive, bivariate, and multivariate analyses. The first step includes the description of all variables in terms of percentages, means, and standard deviations. These variables were clustered in four groups: a) demographic variables, including gender, age, educational level and nationality; b) cocaine consumption pattern, including weekly amount of cocaine consumed, years of cocaine dependence, and main cocaine route of administration; c) other substance dependencies, including cannabis, opioids, benzodiazepines, alcohol, and tobacco dependence; and d) personality disorders (Antisocial Personality Disorder and Borderline Personality Disorder). The second step consisted of analyzing bivariate associations between each aforementioned variable and CIP using Student’s t test and Chi-square tests for quantitative or categorical variables. The third step was a multivariate analysis. A logistic regression analysis was performed, age, sex, consumption of alcohol, benzodiazepine, amphetamines, ecstasy, opiates, and cannabis were included as independent variables. Nagelkerke R square was used to calculate the effect size. The five ZKPQ personality variables were also included under a conditional entrance procedure. The dependent factor was the dichotomy status of patients (with or without psychotic symptoms). All statistical hypotheses were two-tailed. SPSS, version 20, for Windows was used for all analyses.

## Results

CIP was reported for 65.4% of the patients. Some PD was detected in 46.8% % of the sample. The description of the sample is provided in Table 1.

**Table 1:** Descriptive statistics for the study sample.

Gender	Males	(n = 179) 77.5%
Age	M (SD)	36.3 (7.5; range: 19–59)
Education	Primary	(n = 114) 49.3%
	Secondary	(n = 98) 42.3%
	High	(n = 19) 8.5%
Civil status	Single	(n = 90) 39.6%
	Married/living together	(n = 74) 32.6%
	Divorced	(n = 60) 26.5%
	Widowed	(n = 3) 1.3%
Personality Disorders		
	Borderline	(n = 38) 16.5%
	Antisocial	(n = 46) 19.9%
	Avoidant	(n = 8) 3.5%
	Obsessive Compulsive	(n = 11) 4.8%
	Passive Aggressive	(n = 4) 1.7%
	Schizotypal	(n = 3) 1.3%
	Paranoid	(n = 7) 3.0%
	Dependent	(n = 4) 1.7%
	Depressive	(n = 6) 2.6%
	Histrionic	(n = 4) 1.7%
	Narcissism	(n = 4) 1.7%

**Figure 2:**
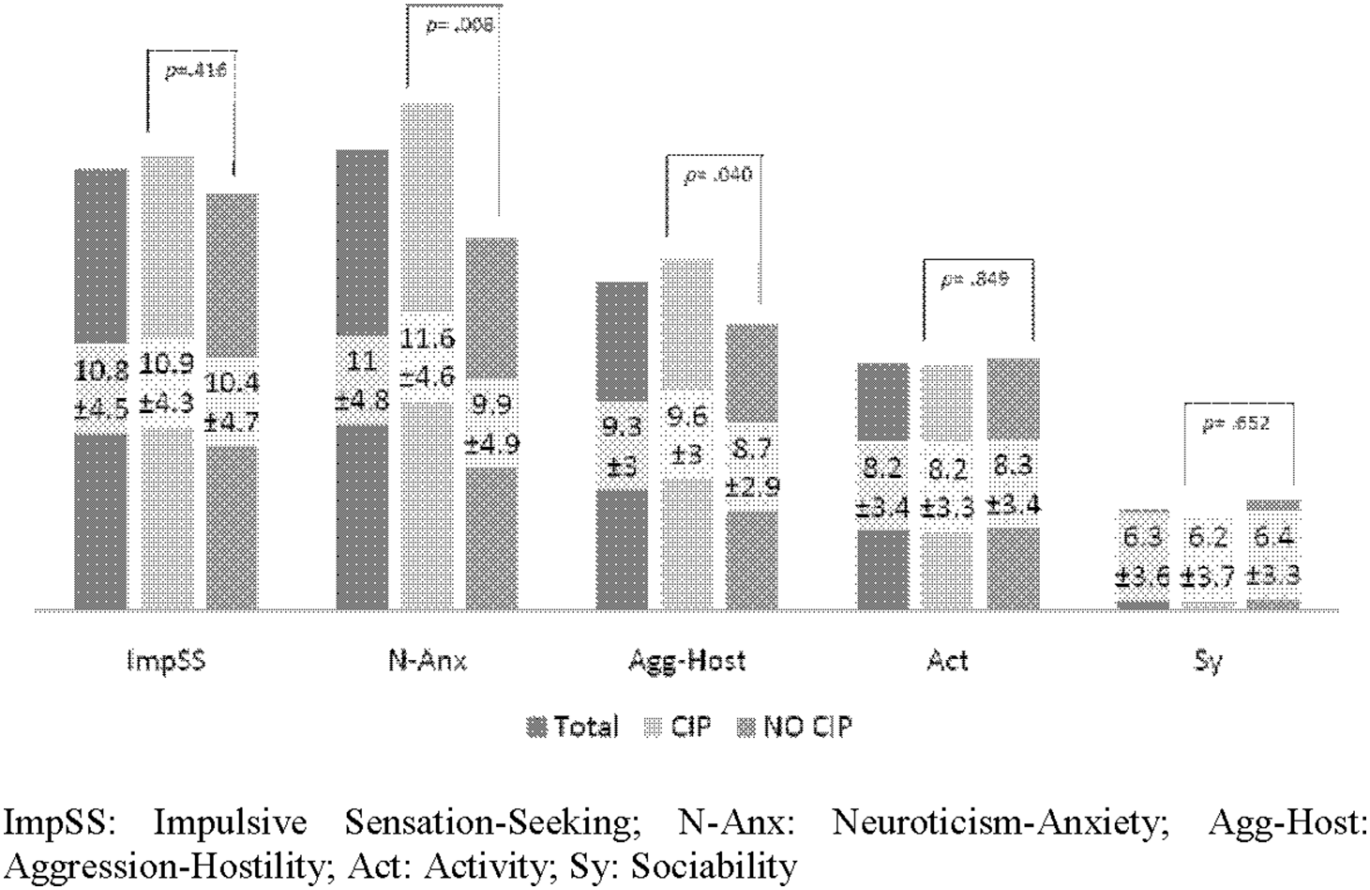
ZKPQ in the total sample and comparison of cocaine-dependent patients with or without cocaine-induced psychosis. ImpSS: Impulsive Sensation-Seeking; N-Anx: Neuroticism-Anxiety; Agg-Host: Aggression-Hostility; Act: Activity; Sy: Sociability.

Means, standard deviations, and *t*-test comparisons are reported in Figure 2. Two personality variables presented a significant effect, N-Anx and Agg-Host (*t*(229) = 2.69, *p* = .008 and *t*(229) = 2.06, *p* = .040, respectively). Regarding the infrequency scale, significant differences were not found among patients with and without CIP (1.65±1.29 versus 1.57±1.12; *t*(229) = .43, *p* = .665).

Patients with psychotic symptoms showed higher scores on both variables than patients without symptoms. In terms of control response, it is important to note that the Infrequency scale did not show a significant discrepancy between CIP and non-CIP patients (Figure 2).

As described above, a logistic regression analysis was performed in order to detect the most personality variables which most differentiate between the presence and absence of psychotic symptoms adjusting for age, sex, consumption of alcohol, benzodiazepine, amphetamines, ecstasy, opiates, and cannabis. The resulting model, presented in Table 2, was significant (Chi Square = 16.13, *p*<.05; Nagelkerke R square = .093). Only N-Anx remained as a significant personality factor (Wald = 7.44, *p*<.05, OR = 1.09, 95% CI 1.02–1.16).

**Table 2:** Multivariate analysis adjusting the effect of the variables significantly associated in bivariate analysis in relation to the presence or absence of CIP.

	Wald	*p*	OR	CI 95% OR
Sex	.17	.679	.86	(.42–1.72)
Age	3.61	.057	.96	(.92–1,00)
Alcohol dependence	.09	.767	.86	(.32–2.32)
Benzodiacepine dependence	.62	.431	.78	(.42–1.45)
Amphetamine dependence	.24	.625	.81	(.34–1.90)
Ecstasy dependence	.28	.594	1.25	(.54–2.89)
Opiates dependence	.00	.957	1.02	(.53–1.96)
Cannabis dependence	2.31	.128	1.71	(.86–3.41)
Neuroticism-Anxiety	7.44	.006	1.09	(1.02–1.16)

Notes: 1 = male, 2 = female. Alcohol, benzodiazepine, amphetamine, ecstasy, opiates and cannabis consumption: 1 = presence, 0 = absence. Consumption variables plus sex and age were included in the model as an adjusting variable (Enter procedure). Personality variables were included using a conditional entrance.

## Discussion

In the present study, neuroticism has been found to be associated with CIP in cocaine-dependent patients, independently of other consumption variables. Previously, neuroticism was associated with increased drug abuse and psychiatric severity and worse outcome [42]. Recently, neuroticism was found to be associated with CIP in the European American population [26] and was also observed to be strongly and independently associated with the first psychotic episode [44], [45]. Some factors could explain this relationship because cocaine-dependent patients with elevated neuroticism tended to use cocaine more frequently [25] and continuously throughout treatment [42]. Both factors, severity and frequency of use, could mediate the association between neuroticism and CIP.

Neuroticism implies more reactivity-anxiety and could reflect excessive physiological responsiveness (or arousability) [56]. Higher trait anxiety was described in patients with CIP compared to cocaine-dependent patients without CIP [57]. From a cognitive approach, neuroticism has been associated with a negative bias in attention, interpretation, recall of information, stressful event generation, and relatively ineffective coping [56]. Finally, from a neurobiological perspective, it has been observed that, in healthy individuals, the central dopaminergic system may play an important role in the neurobiological characteristics of neuroticism [58].

Neuroticism was found to be significantly correlated with the densities of and affected by dopaminergic receptors in both genders [58], [59]. Cocaine is believed to produce psychotic symptoms through increased cortical and subcortical dopaminergic levels, and it is possible to identify an increased synaptic cocaine level both in the cocaine effects and in the positive psychotic symptoms [60]. It could therefore be hypothesized that a dysfunction of the dopaminergic system linked to neuroticism can lead to CIP when a patient is consuming cocaine.

**Figure 3:**
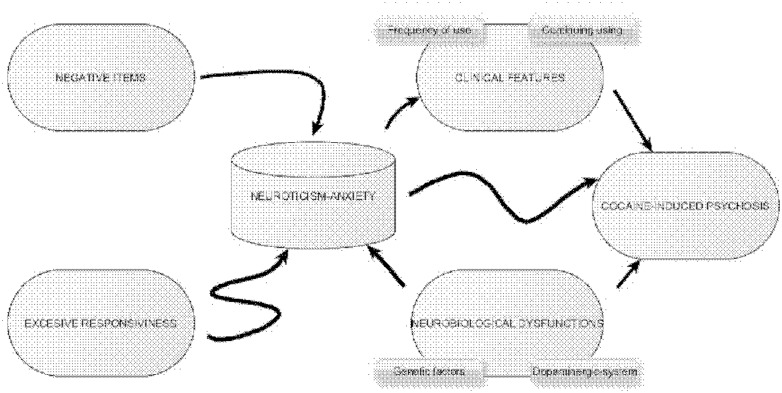
Relationship between Neuroticism and Cocaine-Induced Psychosis.

The relationship between cocaine dependence and CIP is very complex [61]. Furthermore, the correlation between neuroticism and CIP is positive but could be explained by a number of different factors (Figure 3). Neuroticism and associated anxiety could be related both to the influence of negative inputs and to an excessive arousal and low stress tolerance (excessive responsiveness). as well as a dysfunction of dopaminergic origin similar to CIP. Neuroticism has also been associated directly and independently to the occurrence of CIP and indirectly to the presence of variables related to a predisposition to increased psychotic symptoms such as frequency, amount of consumption, or absence of withdrawal.

In our sample, patients with CIP were found to have higher scores in aggression-hostility. In a previous study, in the first episode of psychosis, patients presented higher scores in aggression-hostility than healthy participants [44]. Also, in cocaine-dependent patients, aggression-hostility was associated with greater drug abuse and psychiatric severity [43]. Furthermore, CIP has been associated with agitated behavior and aggression [10], [12], [15], legal problems [20] and, in the same studies, with antisocial personality disorder [6], although this association is controversial [9], [23].

A common origin of the development of behavioral disorders and psychotic symptoms has been proposed [10]. However, there is a lack of studies specifically demonstrating the association between CIP and hostility, and this association was not found in the multivariable analysis, suggesting that, this relationship should be studied in more depth.

This is the first description of the relationship between personality dimensions and CIP using ZKPQ. The strong points of this study are the use of a systematic evaluation process and semi-structured interviews providing high diagnostic reliability. Moreover, the size of the samples in in this study was larger than what is standard in most CIP studies to date. There is only one study describing the association between CIP and neuroticism [26].

Some limitations of the study should be noted. We included cocaine-dependent patients seeking treatment and who were willing to participate in the evaluation process in the analyses, and as such, may not be representative of the entire cocaine dependent population. Nevertheless, it is expected that patients who seek treatment in an outpatient drug clinic would be more dysfunctional than cocaine-dependent consumers not seeking treatment. Research focused on street drug users found higher severity/greater dysfunction in those in clinical settings versus those not receiving treatment [62]–[64]. Because of this, it is possible to hypothesize that drug users not in treatment should present lower neuroticism scores. Another limitation is that this type of study is based on patients’ retrospective self-report and could be associated with diagnostic errors of the symptoms. However, several studies have shown good results with self-report in drugs settings [65]. Finally, it would be interesting to compare the influence of neuroticism on increasing the risk of psychosis symptoms in cocaine-dependent patients in contrast to the influence of neuroticism on the risk of presenting psychosis in general population.

## Conclusions

The relationship between personality traits (such as neuroticism) and the presence of psychotic symptoms is very complex. It is possible that it was a factor in modulating or influencing the risk of a psychotic experience after cocaine consumption (CIP). We detected high scores in neurotic traits using the ZKPQ in patients with a history of CIP. Neuroticism was found to be associated with psychotic symptoms in cocaine-dependent patients, independently of other consumption variables.

A psychotherapeutic approach in patients with high scores in neuroticism should be used in order to develop strategies to control the negative aspects of neuroticism (impulsivity, negative feelings…). This could have a positive effect on the prevention of psychosis symptoms due to cocaine abuse. Personality dimensions should be evaluated in cocaine-dependent patients. Higher scores in neuroticism should require an evaluation of the risk of CIP in order to warn the patients about the risks of developing psychotic states.
